# 

**Published:** 1889-02

**Authors:** 


Receipted for 1888—Dr. J. W. Baker, J. T. Hunter, J. T. Cleveland, H. S. Lawson, T. F. Burnett, E. K. Bozeman, J. M. Stansill, W. H. Boykin, B. E Clark F. C. Davis L. S. Brownlee, T. P. Oliver, W. J. Hill

For 1889—Lib. Sur. Gen'l., A W Irwin, E A Anderson, P C Tircuit, A A Latta, R C McCann, H L Sutherland, J H Boggan, J A Gordon; to July, J W Baker T R Bennett, J E Frippe, W W Leak, to Octcber, Robt. Middleton for 87.

